# Advancing adoptability and sustainability of digital prediction tools for climate-sensitive infectious disease prevention and control

**DOI:** 10.1038/s41467-025-56826-6

**Published:** 2025-02-14

**Authors:** Dung Phung, Felipe J. Colón-González, Daniel M. Weinberger, Vinh Bui, Son Nghiem, Cordia Chu, Hai Phung, Nam Sinh Vu, Quang-Van Doan, Masahiro Hashizume, Colleen L. Lau, Simon Reid, Lan Trong Phan, Duong Nhu Tran, Cong Tuan Pham, Kien Quoc Do, Robert Dubrow

**Affiliations:** 1 School of Public Health, The University of Queensland, Brisbane, Queensland Australia; 2 Queensland Alliance for Environmental Health Sciences, The University of Queensland, Brisbane, Queensland Australia; 3 Data for Science and Health, Wellcome Trust, London, United Kingdom; 4 Department of Epidemiology of Microbial Diseases, School of Public Health, Yale University, New Haven, United States of America; 5 Faculty of Science and Engineering, Southern Cross University, Lismore, New South Wales Australia; 6 Department of Health Economics, Wellbeing and Society, Canberra, Australian National University, Canberra, Australia; 7 Centre for Environment and Population Health, Griffith University, Brisbane, Queensland Australia; 8 National Institute of Hygiene and Epidemiology, Hanoi, Vietnam; 9 Centre for Computational Sciences, University of Tsukuba, Tsukuba, Japan; 10 Department of Global Health Policy, Graduate School of Medicine, The University of Tokyo, Tokyo, Japan; 11 UQ Centre for Clinical Research, The University of Queensland, Brisbane, Queensland Australia; 12 Department of Disease Prevention and Control, Pasteur Institute, Ho Chi Minh City, Vietnam; 13 Department of Environmental Health Sciences and Yale Center on Climate Change and Health, School of Public Health, Yale University, New Haven, United States of America

**Keywords:** Viral infection, Climate-change impacts, Epidemiology

## Abstract

Few forecasting models have been translated into digital prediction tools for prevention and control of climate-sensitive infectious diseases. We propose a *3-U* (useful, usable, and used) research framework for advancing the adoptability and sustainability of these tools. We make recommendations for 1) developing a tool with a high level of accuracy and sufficient lead time to permit effective proactive interventions (*useful*); 2) conducting a needs assessment to ensure that a tool meets the needs of end-users (*usable*); and 3) demonstrating the efficacy and cost-effectiveness of a tool to secure its adoption into routine surveillance and response systems (*used)*.

## Abstract (web summary)

Many studies have developed methods for predicting the incidence of climate-sensitive infectious diseases, but implementation of these tools is limited. In this Perspective, the authors discuss how tools should be developed with a view to ensuring adoptability and sustainability.

## Introduction

### Climate-sensitive infectious diseases

The Intergovernmental Panel on Climate Change has concluded with high confidence that climate change has caused and will continue to cause an increased incidence of climate-sensitive infectious diseases (CSIDs), including vector-borne, food-borne, water-borne, and respiratory diseases^1^. Increasing temperature can enhance the transmission of vector-borne diseases by increasing vector survival, feeding activity, and replication rate; increasing the rate of development of the pathogen within the vector; lengthening the transmission season; and expanding the geographic areas suitable for transmission^2,3^. In addition, changes in precipitation can increase vector abundance in context-specific ways^2,3^. A warming climate can also increase the incidence of water-borne and food-borne diseases by accelerating the proliferation of pathogens in their habitats and causing more frequent extreme weather events that facilitate the spread and outbreaks of these diseases^4,5^. Exposure to wildfire smoke, which is increasing due to climate change, may increase the risk of respiratory infections^6,7^. Furthermore, both unusually wet and unusually dry conditions are associated with increased risk of fungal and other respiratory infections in sometimes complex ways^8–10^, and meteorological factors, including temperature, humidity, and precipitation, influence the seasonality of viral respiratory infections^11^.

### Opportunities and barriers to develop prediction tools for CSIDs

Digital technology could facilitate the incorporation of climatic big data into disease surveillance systems to enable them to serve as early warning systems for CSIDs^12^. Climate-informed statistical models can be used to develop digital tools to forecast CSID outbreaks at different spatiotemporal resolutions^13^. Such tools, integrated into surveillance systems, could provide the lead time needed for public health professionals to thwart predicted outbreaks by proactively implementing preventive measures, in partnership with communities^12–14^. Such early warning systems are especially important to implement in low-and middle-income countries (LMICs)^3,15^, which suffer the worst impacts of climate change, have the highest incidence of CSIDs like dengue and malaria, and have the least adaptive capacity to address these impacts due to limited resources.

Numerous studies have developed climate-informed forecasting models for CSIDs. However, only a small proportion of these models have been translated into user-friendly digital tools for incorporation into routine surveillance systems^13,16^. Furthermore, the relatively few developed tools rarely have been used in the field for disease prevention by public health practitioners. A comprehensive landscape mapping review published by the Inter-American Institute for Global Change Research identified 37 existing climate-informed CSID modelling tools that were transparently described and validated, named, and accessible^13,16^. Thirty of these tools focussed on vector-borne diseases. Twenty of the tools had either been presented as an accessible product or used in an implementation setting; however, only one-quarter of the tools had interfaces legible to decision-makers. The review did not attempt to determine whether the tools were being actively used to inform decision-making.

The Inter-American Institute for Global Change Research landscape mapping review included interviews with researchers and policy stakeholders that revealed several important barriers to the use of climate-informed CSID models as forecasting tools by public health practitioners^13,16^. One key barrier was an inadequate collaboration among modellers, tool developers (i.e., software engineers), and decision-makers to translate models into practical tools that would be adopted by decision-makers. A second key barrier was insufficient training for practitioners who would use the tools. These barriers pointed to the need for the co-creation of practical, user-friendly, sustainable tools through multi-sectoral collaboration and local training and capacity building. Finally, there is a paucity of compelling evidence on the efficacy and cost-effectiveness of prediction tools for reducing the incidence of CSIDs. Such evidence is needed to make a case for public health authorities to adopt these tools.

## Goal of this perspective

Based on our experience in research and practice, our multidisciplinary team of experts from disciplines including medicine, public health, epidemiology, infectious disease modelling, climate science, sociology, economics, health services and management, and information and communication technology, believe that for CSID prediction tools to be sustainably adopted into routine surveillance systems, they must first be shown, according to the concept of use^17^, to be *useful, usable*, and effectively *used* for disease prevention and control.

**Fig. 1:**
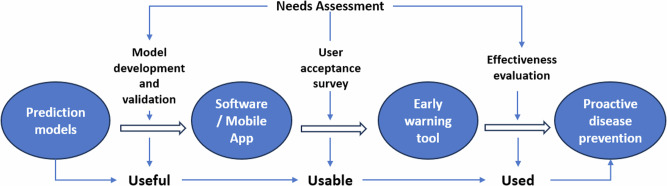
The research framework based on the 3-U principles^17^. Each blue oval represents a stage in the development of an early warning system. Methods to ensure that the early warning system is useful, usable, and used are applied at specific stages of the development process. In addition, a needs assessment should be conducted for each stage of the project.

The concept of use, which we borrowed from the field of product design, has three contextual principles: *useful*, *usable*, and *used*^17^. A product is *useful* if it allows the users to accomplish a specific purpose. However, it may not be used if it is not *usable*, meaning that the product is readily understandable, user-friendly, and can be used in a simple, convenient, efficient, engaging, and effective manner. Furthermore, a product may be both useful and usable but still not *used* if, for example, it does not meet defined needs and cost-effectiveness so that it is adopted by potential users. In this Perspective, we propose a comprehensive research framework for advancing the adoptability and sustainability of CSID prediction tools, based on the concept of use and the *3-U* principles (Fig. 1).

## How to make the tool *USEFUL*

A *useful* tool should be based on a CSID-specific model that predicts the incidence or an outbreak of the CSID with a high level of accuracy and with enough lead time to permit effective proactive prevention efforts. Mechanistic models, based on first principles, simulate biological processes involved in transmission dynamics, whereas empirical models are based on observed statistical associations between predictors and the CSID outcome. Many prediction models incorporate both mechanistic and statistical components^18^.

### Model development

A prerequisite for adequate model development is the availability in the target geographic location of high-quality, long-term datasets for the daily, weekly, or monthly incidence of the CSID of interest; climate data, such as daily mean air temperature and daily precipitation; and other key predictors, such as population density, land cover, and socioeconomic variables. An important limitation is that some or all of these data may be of insufficient quality (e.g., paper rather than electronic records) or not available at all, especially in LMICs and other low-resource settings, in which case development of a prediction model and subsequent tool may not be possible. Thus, before a project to develop a digital prediction tool for CSID prevention and control can be launched, consultations with data providers are essential to understand the existence, features, strengths, and limitations of these datasets. Furthermore, a systematic review could be conducted to identify previous modelling approaches for the CSID of interest, their strengths and limitations, and climate and non-climate predictors identified in previous studies^19^. Then, CSID outcome and predictor datasets at the appropriate spatiotemporal resolution need to be identified and access secured.

It would be valuable for public health authorities to incorporate forecasting uncertainties into their decision-making^20,21^. Therefore, approaches that yield probabilistic outcomes are most useful. For instance, spatiotemporal models fitted using a Bayesian framework enable quantification of the probability that an outbreak may occur at a specific time and location^22^. While a number of machine learning methods have recently been developed for forecasting, they often do not yield uncertainty estimates^23^. The ideal model should provide accurate disease forecasting at a small enough spatial scale and a long enough lead time to make it feasible for resource-constrained public health departments to proactively implement the measures needed to prevent a CSID outbreak in time to make a difference. Finally, previous studies have found that ensemble models, which aggregate multiple independent base models, generally outperform the individual models^24,25^, suggesting that ensemble models may be favoured for CSID forecasting.

### Model validation

**Fig. 2:**
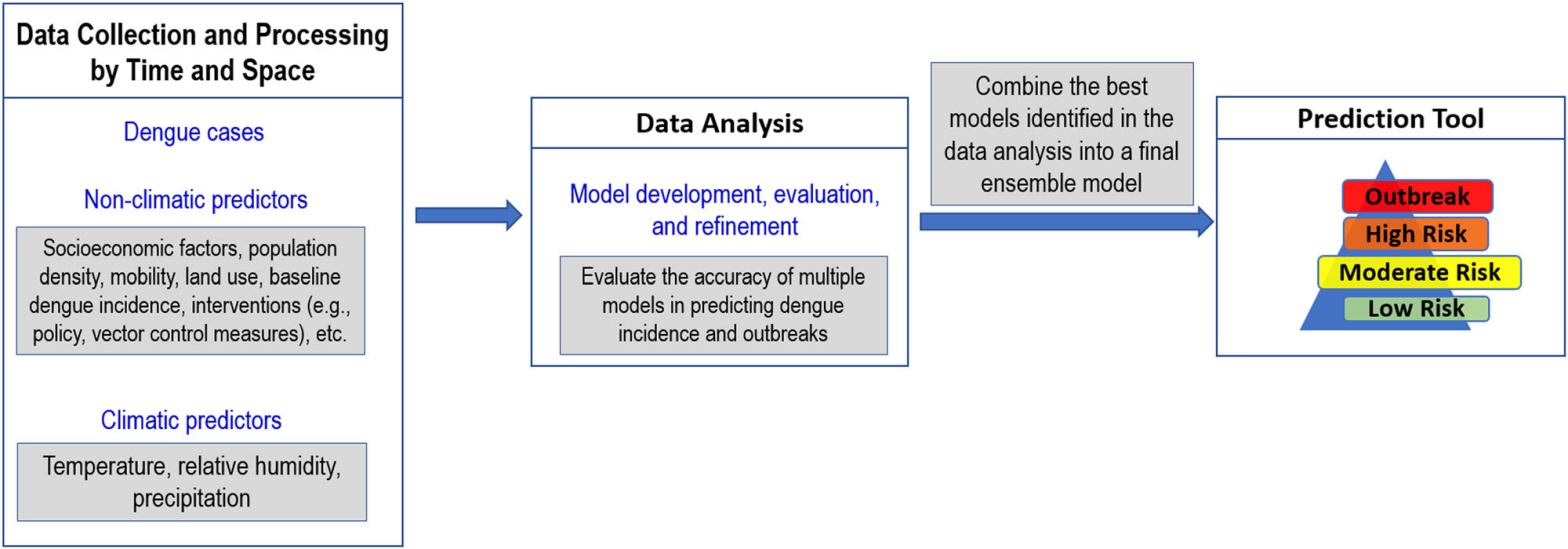
Framework for developing and validating a dengue prediction model. Each box represents a step in the process of developing and validating a prediction model. The grey boxes provide details on the methods and data used for model development and evaluation. The figure in the “Prediction Tool” box represents a colour code for a warning message.

A validation process, which evaluates the accuracy of a model in predicting disease incidence and outbreaks, is essential for selecting the optimal model(s). Time series cross-validation is a general tool for assessing the predictive ability of a model^26^, in which a model’s predictive performance after a training period is tested by comparing predicted and observed values using the leave-future-out cross-validation technique^27^. To validate the prediction of CSID incidence, three inter-related metrics can be used in concert^28^: (i) *Sharpness* examines the variance of the predictive distribution at each time point; (ii) *Bias* tests whether a model systematically over- or under-predicts; and (iii) *Continuous Rank Probability Score* (CRPS) is a summary measure that rewards sharp, unbiased forecasts. CRPS is similar to a mean absolute error but uses the full probability distribution of the forecasts instead of a point estimate, making it relevant for Bayesian prediction models^22^. It is desirable for forecasts to have a low CRPS (CRPS has no upper bound, with zero indicating a perfect forecast) and to be sharp (i.e., for the predictive distribution to have low variance) and unbiased. Validation is also needed to assess the accuracy of a model for predicting CSID outbreaks (as opposed to incidence), which occur when the number of observed cases exceeds a location-specific epidemic threshold. The *Brier score*^29^, used to measure the accuracy of probabilistic forecasts for predicting events, is commonly used for validation of outbreak predictions of Bayesian models. An example of model development and validation for dengue forecasting is shown in Fig. 2. It is possible that a model that predicts CSID incidence and outbreaks far enough in advance to be useful will not be identified, in which case the project should be abandoned.

## How to make the tool *USABLE*

A *usable* tool is user-friendly, simple, and meets the needs of end-users (e.g., public health practitioners) for CSID prevention and control. Such a tool could be a web-based software system with a mobile app interface based on the outputs of the prediction model. The tool should allow users to input data, receive alerts, and access real-time information conveniently from anywhere. Ideally, a usable tool would be integrated into a routine surveillance system for the CSID in question, based on datasets that are automated and updated in real-time by existing systems and available for future use in the developed tool for routine forecasting. It is important to recognise that in many settings (especially LMICs and other low-resourced settings) routine surveillance systems are rudimentary or do not exist and that existing systems (which vary widely in type, quality, human resources, and financial capacity) may not be automated, updated in real-time, and/or available for future routine use. In many instances, after these usability factors are carefully assessed, a decision may be made that substantial capacity building would be needed as a prerequisite to tool development.

### Needs assessment

Furthermore, to ensure that the tool meets the needs of end-users, once a decision is made to proceed with tool development, a needs assessment (NA) should be conducted for each stage of the project. The NA, which should incorporate assessments of community and public health system assets and technology acceptance of potential tool users, should engage a wide range of stakeholders, including decision-makers, public health practitioners, data providers, researchers, other technical experts, and community leaders.

For the early model development and validation stage of the project, the NA should identify the needs to ensure that the tool is appropriate and acceptable to relevant stakeholders. For example, public health practitioners may have a well-established definition for an outbreak of the CSID in question. If this is the case, ideally the model should adhere to this definition. However, if the model developers find that another definition would be more useful for forecasting, a negotiation process between the research team and the practitioners should ensue to come up with an outbreak definition that both allows for optimal forecasting and is acceptable to the practitioners.

For all stages of tool development, the NA should assess the requirements for the tool to be effectively used in the surveillance system and routine prevention practices. For example, when the tool predicts an outbreak, what will the proactive intervention to prevent the outbreak look like? This will depend on factors such as the financial and human assets available to the public health system, as well as practitioners’ beliefs about the effectiveness of alternative intervention strategies. If the proactive intervention might involve community mobilisation, then assessing community assets that could aid in the mobilisation, such as faith-based organisations and media outlets, would be relevant. Again, a negotiation between the research team and the practitioners may be needed to settle upon the characteristics of the proactive intervention. For the project’s final stages and the post-project stage, the NA should evaluate the need for sustaining and expanding the use of the tool going forward. This often will be a question of assessing financial and human assets as well as public health system priorities and needs.

**Fig. 3:**
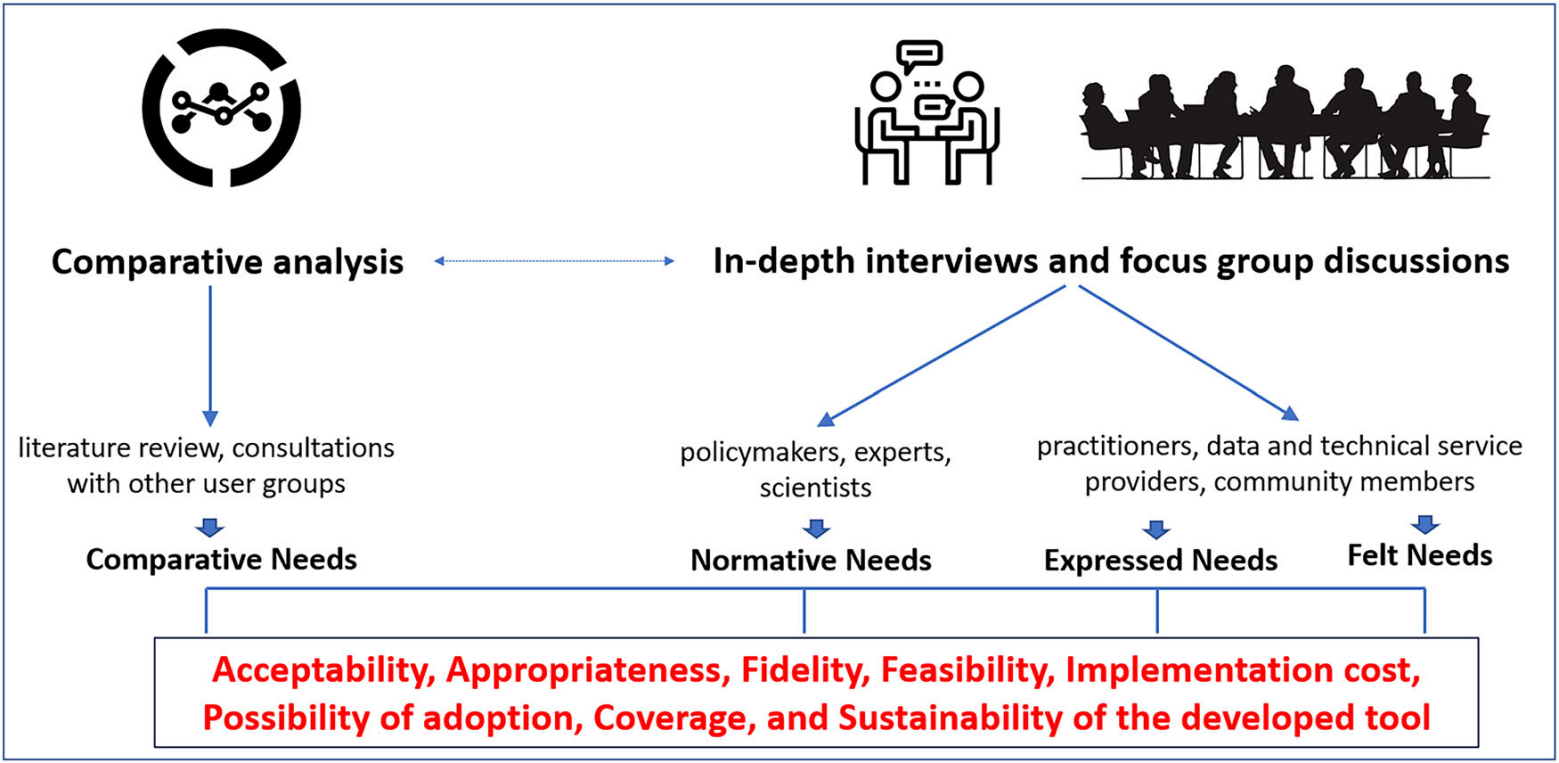
Needs assessment framework. This framework is used to identify needs related to integrating a tool into a routine surveillance system and preventive practices. The top row of the figure represents the needs assessment methods. The second row describes the data collection process and research participants included in each needs assessment activity. The third row shows the corresponding types of needs identified by each needs assessment method. The final box shows the findings of the needs assessment.

Bradshaw’s widely-used concept of four types of needs, as stated in his seminal 1972 *“Taxonomy of Social Need”*, can provide a foundational framework for an NA and categorising and understanding the complexity of needs^30^. It has been used to inform social policy design, service allocation, and resource distribution. *Comparative needs* are defined objectively and are assessed by comparing the health status of populations and the health services available to them. *Normative needs*, defined by experts, set a standard against which the tool and its use should be evaluated. *Expressed needs* are defined by what products or services a community uses. *Felt needs* are defined by what members of the target population state their needs and desires to be. An NA process for tool development can be based on a literature review and consultations with other user groups (*comparative needs*), as well as in-depth interviews or focus group discussions with 1) policymakers, experts, and scientists (*normative needs*) and 2) practitioners, data and technical service providers, and community members (*expressed needs* and *felt needs*) (Fig. 3).

A usable tool is easy to use, compatible with existing systems and technologies, and aligns with the users’ skills and capabilities. Previous studies have shown that the “fit” between information technology (IT) and routine clinical practices “will lead intended end-users to accept or reject the IT, to use it or misuse it, to incorporate it into their routine or work around it”^31^. This is why the assessment of technology acceptance of potential users is a vital component of the NA. Various technology acceptance frameworks that consider determinants of technology use, such as perceived usefulness, perceived ease of use, social influence, and habit may be used to facilitate this assessment^32–34^.

NAs have limitations. First, *felt* needs vary among individuals and cultures, leading to potential biases depending on the perspectives of the key informants. Thus, involving diverse and representative stakeholders is crucial^35^. Second, not all *expressed* needs are communicated due to barriers such as lack of access. Triangulating multiple sources of information can help reveal these hidden needs within the community. Third, because needs may evolve over time, researchers should maintain stakeholder engagement throughout the project and establish feedback mechanisms to capture changing needs. Finally, needs are often complex and conflicting among different stakeholders, which may complicate identifying solutions that address all perspectives. Researchers must prioritise needs that are both actionable and aligned with the broader objectives of the project^36^.

## How to advance the tool *to be USED*

### Efficacy evaluation

Even if an early-warning tool is useful and usable, it still may not be used if there is limited or no evidence of its efficacy and cost-effectiveness for reducing CSID incidence. A randomised controlled trial is the gold standard study design for assessing the efficacy of a public health intervention. Since an early-warning tool is a community-level, geographically based intervention carried out by a public health department, rather than an intervention that targets individual people, a randomised controlled trial of the efficacy of an early-warning tool must randomise geographic units in concert with their associated public health departments. Such a trial is known as a cluster (i.e., group) randomised controlled trial (CRCT), where the unit of randomisation is by necessity, the cluster (in this case, a geographic unit) and not the individual person^37^. CRCTs have been widely used to assess the efficacy of infectious disease interventions^38^, including, for example, a CRCT of community mobilisation for dengue prevention in 150 census enumeration areas in Nicaragua and Mexico^39^, a CRCT of mass drug administration against malaria in 32 villages in The Gambia^40^, and a CRCT of a community-led total sanitation intervention with a focus on improved household toilets to reduce diarrhoea incidence in children in 48 villages in Ethiopia^41^.

A prerequisite for conducting a CRCT to assess an early warning tool would be a geographic region consisting of sub-regions (i.e., districts) served by a public health department(s) that has (have) the willingness and capacity to implement a proactive intervention when the early-warning tool predicts an outbreak. Clusters would be randomised to an intervention arm and a non-intervention (control) arm. To determine the number of clusters to be randomised, a target reduction in CSID incidence (e.g., 25%) in the intervention arm compared to the control arm needs to be set, and then a sample size calculation needs to be conducted to ensure sufficient statistical power. This calculation needs to take into account both the number of clusters and the number of individuals within clusters due to a phenomenon known as within-cluster correlation; i.e., individuals within clusters tend to be more like each other than individuals in other clusters with respect to the probability of the outcome^42^. Statistical power is inversely proportional to the degree of within-cluster correlation.

**Fig. 4:**
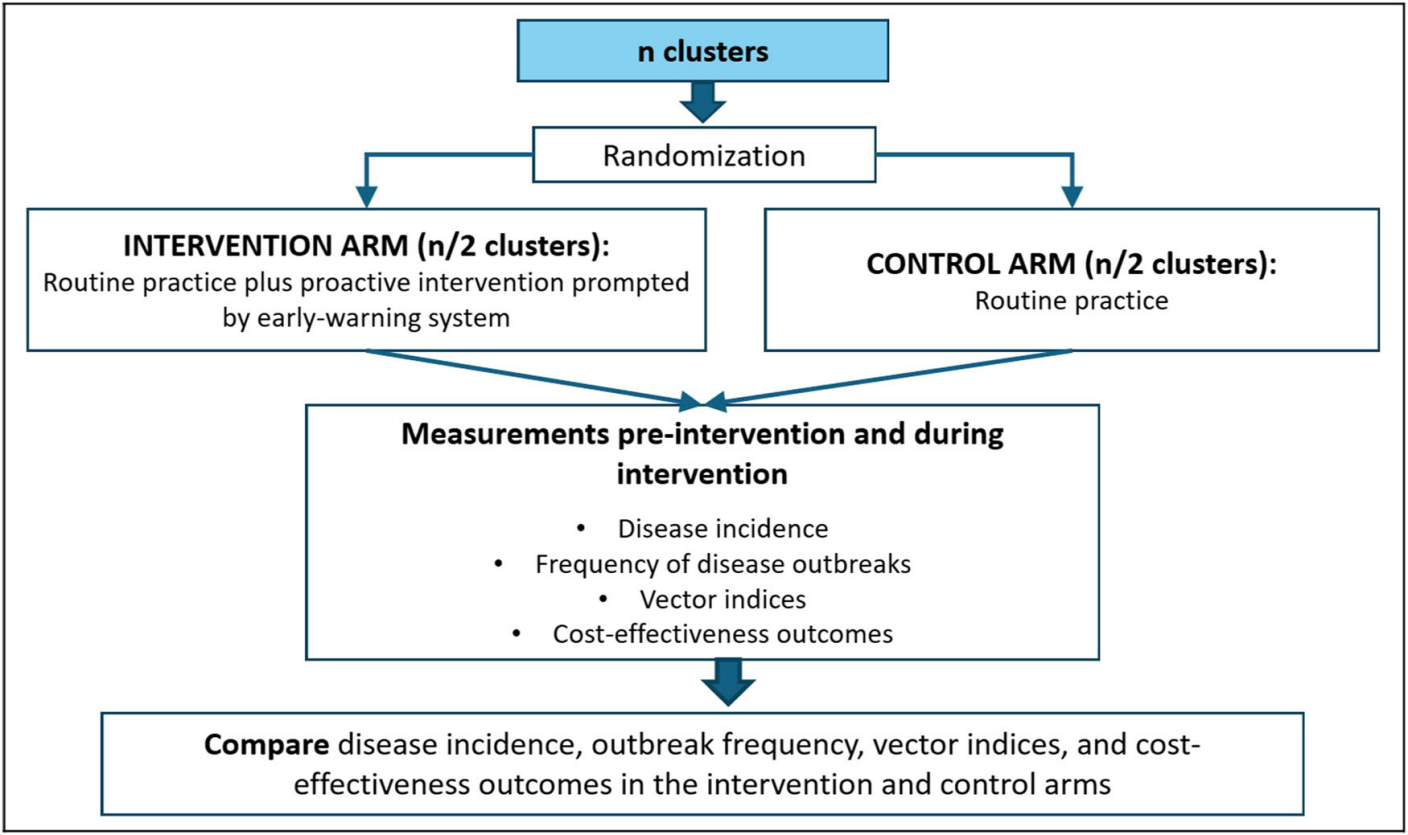
Framework for a cluster-randomised controlled trial (CRCT). Here, we illustrate a CRCT to evaluate the efficacy and cost-effectiveness of a prediction tool for a vector-borne CSID. Clusters are randomly assigned to the intervention arm and the control arm. The clusters in both arms follow routine disease control procedures. The clusters in the intervention arm also receive a proactive intervention prompted by the prediction of an outbreak by the early warning system. Measurement of disease incidence and outbreaks, vector indices, and cost-effectiveness are conducted in all clusters before and during the intervention to compare outcomes in the two arms.

An example of a CRCT for a vector-borne CSID is illustrated in Fig. 4. The non-intervention (control) clusters would conduct routine prevention practices, as promulgated by public health authorities, for the CSID in question. Intervention clusters also would conduct routine prevention practices, but, in addition, if the early-warning system predicts an outbreak, they would conduct a set of proactive preventive interventions prescribed in the study protocol. Primary outcome measures could be disease incidence and frequency of outbreaks, and secondary outcomes could be entomological indices as determined by vector monitoring.

### Cost-effectiveness evaluation

In addition, a cost-effectiveness analysis that generates *value-for-money* evidence should be incorporated into the trial^43^. The key outcome would be the *incremental cost-effectiveness ratio* (ICER), that compares the incremental cost change for disease surveillance and the incremental outcome cost change (e.g., disability-adjusted life years [as determined by society’s willingness to pay], number of hospitalised CSID cases). The tool would be considered cost-effective if the estimated ICER were less than one.

### Limitations of cluster-randomised controlled trials

Although, by necessity, the efficacy of a CSID early warning system needs to be assessed at the level of geographic units, CRCTs have limitations. First, if the within-cluster correlation is high, then a large number of clusters may be needed for sufficient statistical power, which could be expensive and difficult to implement. Second, despite randomisation, CRCTs may be subject to confounding when the number of randomised clusters is relatively small, such that the intervention and control groups may not be comparable with respect to confounders simply due to chance^37^. This limitation could be addressed by conducting stratified randomisation of clusters on important known confounders, such as baseline CSID incidence rate, and by adjusting for confounders in the analysis.

Third, there is the possibility of cross-contamination of intervention and control clusters due to factors such as the movement of people or vectors (in the case of a vector-borne CSID) across clusters, which would make intervention and control clusters effectively more alike and, therefore, bias efficacy estimates toward the null^38,44^. This limitation could be addressed by creating geographic buffer zones between intervention and control clusters, by only using the central part of each cluster in the data analysis (effectively creating geographic buffers), or by using clusters of large geographic size to reduce the impact of cross-cluster movement of people or vectors (in the case of a vector-borne CSID)^38,44^.

Furthermore, the possibility of cross-contamination could be avoided completely by using a quasi-experimental, nonrandomized pre/post-intervention study design instead of a CRCT^45^. In this design, the intervention is implemented in a defined geographic area and the incidence of the CSID being studied is compared before and after the intervention is implemented; without a control geographic area, there is no contamination issue. However, abandonment of a randomised design opens up the possibility of severe confounding as there are many factors, both climatic and non-climatic, that could affect the incidence of a CSID between the pre-intervention and post-intervention periods apart from the intervention itself ^45^. For example, for reasons that are not well understood, between 2023 and 2024 there was an unprecedented increase in probable dengue cases in Brazil – from 1.65 million in the entire year of 2023 to 6.56 million in the first 45 epidemiological weeks of 2024^46^. Thus, it often is preferable to risk bias toward the null in a CRCT versus risking severe confounding in a nonrandomized pre/post-intervention study.

There are specific barriers to conducting trials in LMICs, including low levels of financial and human capacity, ethical and regulatory system obstacles, poor research environment, operational barriers, and competing demands on potential research, practice, or community partners^47^. Finally, it is possible that a CRCT will have a null result, which would indicate that the early warning tool should not be implemented.

## How to make the tool *SUSTAINABLE*

The goal of sustainability of the prediction tool must be considered prior to, during, and after the project.

### Pre-Project

The project should engage stakeholders, including decision-makers, researchers, local, regional, and national public health practitioners, data providers, software developers, other technical experts, and community representatives in designing the project to ensure a sense of ownership and that its desired outcomes are consistent with the needs of the health sector and the community.

### During the project

NAs should be conducted for all phases of the project to ensure the appropriateness and acceptability of the tool to the relevant stakeholders. The CRCT should be designed as a pragmatic trial that ensures alignment with routine practices of the public health departments at various administrative levels (e.g., local, regional, national), including integration of the tool into the existing surveillance and response systems without adversely interfering with current practices.

### Post-project

The following activities would ensure post-project use of the tool:

- Adoption/endorsement of the tool by public health departments, ideally including at the national level (i.e., the Ministry of Health). Once the efficacy and cost-effectiveness of the prediction tool have been established, the project should implement communication activities (technical workshops) to promote such adoption.
- Capacity-building through training-of-trainers (public health practitioners) to advance implementation of the tool beyond the intervention districts in the trial. The trainers would train practitioners in other districts of the country once the tool has been adopted.
- Collaborating with public health departments to integrate monitoring and evaluation of the tool into the routine practices of the CSID surveillance system. Monitoring and evaluating the ongoing effectiveness of the tool in maintaining a reduction of CSID incidence should be funded by regular public health funding schemes.
- To maximise the usefulness, reach, and reproducibility of the project, particularly in LMICs, release the study design and research outputs along with guidance documents via open source. The modelling software tool could be deposited in a version control platform like GitHub. Guidance documents would describe details of the research, including methods, instruments, procedures, statistical analysis, and computer code. In addition, sample datasets, along with a user’s guide that would enable researchers to use the developed software effectively should be published. This would help researchers reproduce the project and develop early warning tools for CSID adapted to their particular settings.

## Conclusion

Although there is a high level of interest in developing prediction tools for CSID prevention, how to ensure the actual use of these tools in routine surveillance systems is still an open question. In 2005, the World Health Organisation published a conceptual framework for developing early warning systems for CSIDs^48^. The publication focused on how to develop a “useful” (our terminology, not theirs) early warning system and performed a detailed evaluation of candidate CSIDs. Although it advocated for including health policymakers in all stages of system design and for rigorous evaluation and cost-effectiveness analysis, it did not provide detailed guidance on developing a “usable” and “used” early warning system.

In this Perspective, we recommend a 3-U framework to promote the usefulness, usability, and actual use of prediction tools, as well as approaches to ensure that the tool is sustainable. We hope our recommendations will help policymakers, researchers, public health practitioners, and donors better formulate projects to advance the adoptability and sustainability of early warning tools for CSID prevention and control. As the incidence of CSIDs continues to increase due to climate change, integrating early warning tools into routine practice will represent a crucial climate change adaptation measure. This will be especially challenging in LMICs and other low-resource settings, where these measures are most needed.
